# The Advanced Behavioral Intervention Design in Dementia Care Training Program: Insights and Outcomes

**DOI:** 10.1093/geroni/igaf122.1506

**Published:** 2025-12-31

**Authors:** Dana Urbanski, Allycia Wolff, Manka Nkimbeng, Emily Mroz, Maria Quiñones-Cordero, Quinton Cotton, Mayra Sainz, Joseph Gaugler

**Affiliations:** Indiana University Bloomington, Bloomington, Indiana, United States; University of Minnesota, Minneapolis, Minnesota, United States; University of Minnesota School of Public Health, Minneapolis, Minnesota, United States; Emory University, Atlanta, Georgia, United States; University of Rochester School of Nursing, Rochester, New York, United States; University of Pittsburgh, Pittsburgh, Pennsylvania, United States; Emory University, Atlanta, Georgia, United States; University of Minnesota, Minneapolis, Minnesota, United States

## Abstract

Non-pharmacologic behavioral interventions are increasingly recognized as essential to address the needs of people living with dementia and their family caregivers. However, university programs are not organized around providing the training needed to prepare early-career investigators to develop, test, and refine such interventions. The Advanced Behavioral Intervention Design in Dementia Care (ABIDDC) program addresses this gap by 1) Delivering an advanced curriculum in dementia care intervention science; 2) Establishing a robust, structured peer mentoring program to support a cohort of early-career investigators in dementia care science; and 3) Leveraging resources at the University of Minnesota (e.g., Center for Healthy Aging and Innovation, aging-related special interest groups, idea-hatching and grant review workshops) to enrich training in dementia care intervention science. This presentation will summarize the key components and outcomes of the ABIDDC during its first two years. We will discuss the ABIDDC curriculum, peer mentoring program, and plans for collaborative projects. Additionally, we will highlight how the ABIDDC facilitates engagement with other programs that offer pilot funding and professional development in dementia care intervention science, such as the Resource Centers for Minority Aging Research Center and the National Institute on Aging IMbedded Pragmatic Alzheimer’s disease (AD) and AD-Related Dementias Clinical Trials Collaboratory. Preliminary outcomes related to trainee engagement, research productivity, and career trajectories will also be shared, offering insights into the ABIDDC’s impact. The session will conclude with recommendations for integrating similar training models into other institutions to prepare the next generation of dementia care intervention scientists.

